# Traumatic testicular dislocation with pelvic fracture in a 17 year old adolescent: A case report

**DOI:** 10.1016/j.eucr.2026.103364

**Published:** 2026-02-05

**Authors:** Wondwose Mengist Dereje, Misganaw Degu Worku, Desalegn Kefale Aegash, Rahel Wubayehu Kahaliw, Mesafint Eyayu Tegegne, Biruktawit Abebe Assmamaw

**Affiliations:** a University of Gondar, College of Medicine and Health Sciences, Department of Neurology, Gondar, Ethiopia; b University of Gondar, College of Medicine and Health Sciences, Department of Surgery, Gondar, Ethiopia; c University of Gondar, College of Medicine and Health Sciences, Department of Orthopedics and Traumatology, Gondar, Ethiopia; d University of Gondar, College of Medicine and Health Sciences, Department of Clinical Chemistry, Gondar, Ethiopia

**Keywords:** Testicular dislocation, Trauma, Orchidopexy, Case report

## Abstract

Testicular dislocation is a rare condition in which one or both testes are displaced from the scrotum to an ectopic location and is often overlooked due to associated injuries. A 17-year-old adolescent presented three weeks after a road traffic accident with left inguinal swelling and pain. Initial assessment revealed a pelvic fracture treated conservatively. Examination showed left inguinal tenderness, and ultrasonography confirmed the testis in the inguinal region. Surgical exploration revealed displacement at the inguinal ring. The testis was repositioned and orchidopexy performed. Recovery was uneventful. Trauma patients with inguinal pain or swelling should be evaluated for testicular dislocation.

## Highlights

• Traumatic testicular dislocation is a rare and frequently missed genitourinary injury.
• High-energy blunt trauma, especially motorcycle straddle injuries, is the most common cause.
• Associated injuries often delay diagnosis during the initial trauma assessment.
• Early imaging and careful scrotal examination are critical for detection.
• Timely surgical exploration and orchidopexy prevent torsion, ischemia, and infertility.

## 1 Introduction

Genitourinary injuries account for approximately 10% of all cases of abdominal trauma,^1^ with up to 67% involving the external genitalia.^1^^,^^2^ Testicular dislocation is defined as displacement of the testis outside the scrotum, and the first case was reported by Claubry in 1818.^3^ It is a rare clinical entity that often goes unrecognized due to the presence of concomitant severe injuries, as frequently described in multitrauma patients, particularly motorcyclists.^1^^,^^2^^,^^4^^,^^5^

Traumatic testicular dislocation is most commonly associated with straddle injuries sustained during motorcycle accidents, in which the rider is propelled forward and the scrotum and perineum are forcefully compressed against the fuel tank.^6^ Dislocation may be unilateral or bilateral and can be classified as superficial where the testis is displaced into the superficial inguinal pouch or internal, in which the testis is forced through the external inguinal ring into the inguinal canal or, in rare cases, the abdominal cavity.^7^

***This manuscript was prepared following the CARE guidelines* (**https://www.care-statement.org).

## 2 Case report

A 17-year-old male adolescent presented to our clinic with severe left inguinal pain of three weeks’ duration. The symptoms began following a road traffic accident in which he was a pedestrian struck by a fast-moving three-wheeled vehicle. The vehicle lost balance and overturned onto the roadside. Immediately after the trauma, the patient noted the onset of mild swelling in the left inguinal region, accompanied by localized pain.

**Fig. 1:**
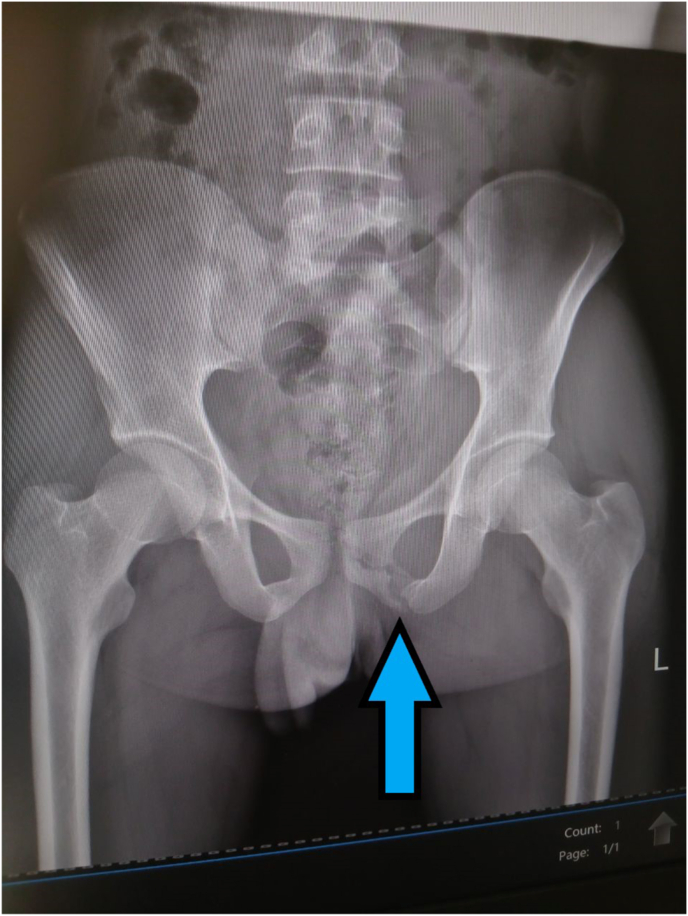
Pelvic radiography showing pelvic ramus fracture (arrow).

He was promptly taken to a nearby health center for evaluation, where pelvic radiography was performed showing pubic ramus fracture (Fig. 1). He was informed that the pain and swelling were secondary to a fracture and that the condition was not considered serious. Consequently, he was prescribed oral analgesics and discharged home. However, beginning on the third day following the injury, the inguinal swelling progressively increased in size, and the associated pain intensified to an unbearable level. Owing to the ongoing war in the region, the patient was unable to access a higher-level healthcare facility and remained at home without further medical intervention for a period of three weeks.

He finally presented to our clinic three weeks after sustaining the trauma. At the time of presentation, he appeared acutely ill and was in severe pain. His vital signs were as follows: blood pressure 100/60 mmHg, pulse rate 102 beats per minute, respiratory rate 19 breaths per minute, temperature 37.1 °C, and oxygen saturation 98% on room air.

On genitourinary examination, there was a tender, swollen left inguinal region. The remainder of the systemic examination was unremarkable.

He underwent laboratory investigations, including a complete blood count, which revealed a white blood cell count of 5 × 10^3^/μL (neutrophils 55%, lymphocytes 37%), hemoglobin level of 13.1 g/dL, hematocrit of 36%, and platelet count of 285 × 10^3^/μL. Urinalysis was normal.

Abdominopelvic ultrasonography demonstrated the left testis positioned at the level of the inguinal ring. In view of the history of trauma and the abnormal location of the left testis which, according to the patient, had been normally positioned prior to the injury testicular dislocation was suspected.

The findings and management options were discussed with the patient and his parents, and surgical intervention was offered. After obtaining informed written consent, the patient was taken to the operating room for testicular repositioning and orchidopexy.

**Fig. 2:**
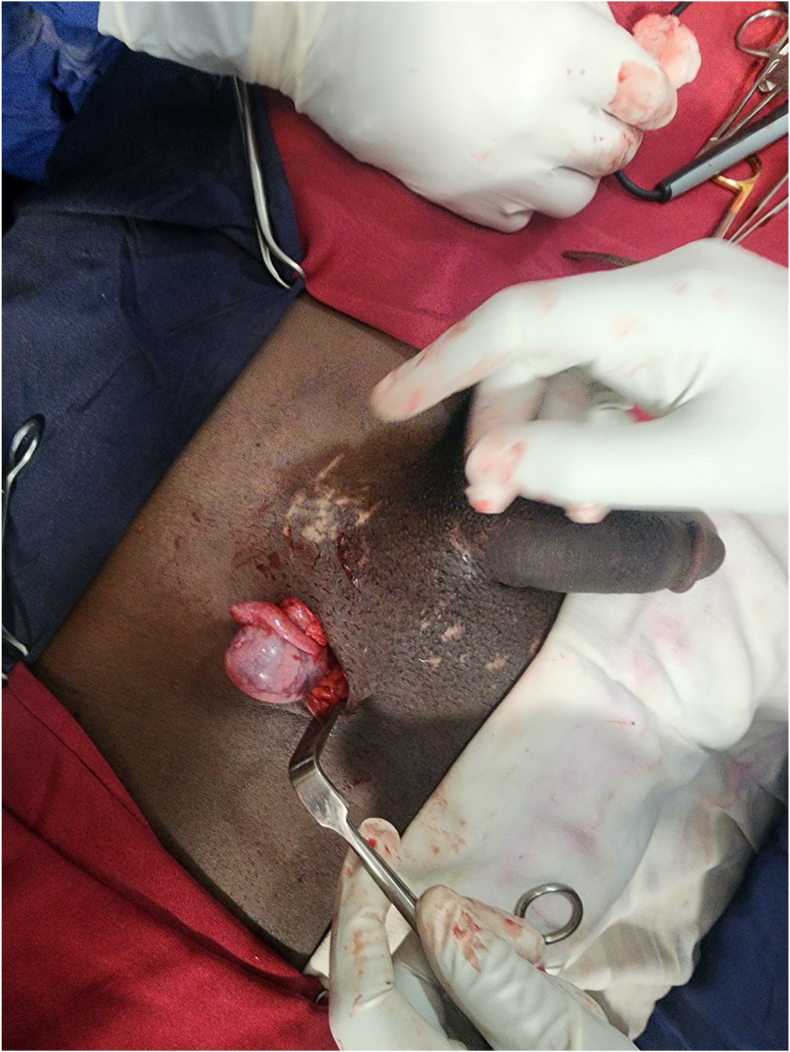
Dislocated left testis to the inguinal canal.

Intraoperatively, the left scrotal sac was found to be empty, and the left testis was dislocated into the inguinal canal (Fig. 2).

**Fig. 3:**
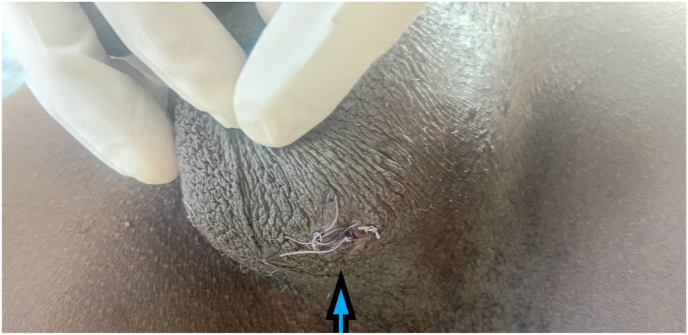
Left testis secured to dartos fascia (arrow).

Inguinal exploration was performed, and the scrotal sac was canalized. The left testis was repositioned to its normal anatomical location within the scrotal sac and secured using the dartos fascia (Fig. 3).

The pelvic fracture had already begun to heal, as demonstrated by pelvic radiography performed at our facility and compared with imaging from his initial presentation; therefore, no intervention was required.

The patient left the operating room with stable vital signs and was admitted for postoperative observation, during which he remained hospitalized for four days. He was discharged on the fourth postoperative day with complete clinical improvement and scheduled for outpatient follow-up.

## 3 Discussion

Testicular dislocation is a rare condition defined as the displacement of one or both testes from their normal position within the scrotum to an ectopic location.^6^ It most commonly occurs following blunt scrotal trauma.^8^^,^^9^ The majority of reported cases result from straddle injuries sustained during high-speed motorcycle accidents, in which the rider experiences a direct impact to the perineum and scrotum against the fuel tank or handlebars.^8^^,^^10^ The wedge-shaped design of the fuel tank directs force toward the groin, forcibly displacing the testes in a superolateral direction.^6^^,^^11^ Although testicular dislocation typically occurs immediately after injury, delayed presentations have been reported, including cases identified up to four days following scrotal trauma.^12^

Although testicular dislocation most commonly occurs unilaterally, bilateral involvement has been reported in approximately one-third of cases.^7^^,^^8^^,^^10^^,^^13^^,^^14^ The dislocated testis may migrate to several ectopic locations. In nearly 50% of cases, the testis is displaced into the superficial inguinal region. Other reported sites include the pubic region (18%), penile shaft (8%), inguinal canal (8%), true intra-abdominal location (6%), perineal region (4%), and crural region (2%).^7^^,^^10^^,^^15^

The final location of a dislocated testis depends on several factors, including the mechanism of injury, the direction and magnitude of the applied force, the presence of underlying anatomical abnormalities, and a brisk contraction of the cremaster muscle at the time of trauma, often followed by secondary cremasteric spasm 16, 17, 18, 19. Diagnosis may be delayed when physical examination of the scrotum and testes fails to reveal clear clinical signs of abnormality.

Although this rare condition can be missed during the primary survey of a trauma patient, it should be specifically evaluated during the secondary survey. Careful scrotal examination as part of a comprehensive physical assessment is essential for early diagnosis, although a scrotal hematoma may occasionally mask the dislocation.^20^

On physical examination, the presence of an empty scrotal sac usually warrants further evaluation. The diagnosis is supported by detection of a tender, firm mass corresponding to the displaced testis, together with an empty hemiscrotum in a patient without a history of orchiectomy or cryptorchidism.^13^

In cases where physical examination findings are inconclusive following scrotal trauma, ultrasonography should be performed. Ko et al. reported a series of nine patients with groin trauma in whom associated testicular dislocations were initially missed in all cases.^11^ Ultrasound can assist in localizing the testis and assessing for capsular or parenchymal injury. Its advantages include the absence of ionizing radiation, lower cost, and rapid availability of results.^12^

In trauma patients, abdominal ultrasonography is often primarily directed at identifying free fluid, and the detection of other pathologies largely depends on the clinician's expertise and level of suspicion. Early communication of suspected testicular dislocation by the clinician is essential to guide the radiologist's or sonographer's interpretation. Few reports address the role of computed tomography (CT) in the rapid diagnosis of penoscrotal trauma. However, as CT has become a routine imaging modality for patients with major trauma and pelvic injuries, early recognition of testicular dislocation in the emergency department is expected to increase. When CT confirms the diagnosis, further evaluation with ultrasonography is frequently unnecessary before surgical management.^18^

Despite this, some consensus statements continue to exclude CT from triage protocols for blunt scrotal trauma.^14^ The differential diagnosis includes undescended testis, retractile testis, and traumatic testicular torsion with a high-riding testis.^7^^,^^11^ Although many authors recommend attempting manual reduction initially in cases involving a normal testis without associated injuries, success is achieved in only about 15% of cases. Contributing factors to failed closed reduction include minor injuries within the spermatic cord layers, edema, and the presence of concomitant injuries.^10^^,^^13^

For example, the presence of associated testicular torsion or rupture contraindicates closed reduction. In such situations, ultrasonography or another appropriate imaging modality should be performed before attempting manual reduction.^11^ Moreover, delayed reduction in postpubertal males has been reported to impair spermatogenesis, usually becoming evident about four months after dislocation.^10^ The major risk of delayed intervention is failure to recognize torsion of the displaced testis, which may lead to ischemia and gangrene of an initially viable testis.^19^

In the present case, the patient presented late, and due to concerns about missed torsion, manual reduction was not attempted. Early surgical exploration and orchidopexy are recommended to evacuate hematomas, repair lacerated tissue, and secure the testis in its normal position. Tai et al. have suggested that surgical exploration of the inguinal and scrotal regions offers advantages over manual reduction, as it allows direct identification of the dislocated testis and simultaneous management of associated injuries.^10^

Surgical exploration eliminates the risk of iatrogenic torsion that may occur during closed reduction, which would otherwise necessitate post-reduction Doppler evaluation to exclude vascular compromise.^11^ Several studies support surgical management, as associated testicular injuries may coexist and manual reduction has a low success rate.^10^ Surgical intervention enables earlier recovery of spermatogenesis, provides prompt pain relief, and reduces overall morbidity. In the present case, the patient achieved marked symptom improvement and was able to ambulate by the second postoperative day.

The standard approach to managing testicular dislocation is fixation of the testis to the dartos fascia.^15^

The main rationale for using a subdartos pouch for testicular fixation is to avoid sutures crossing the blood–testis barrier, which may provoke an autoimmune response and contribute to infertility. Suture fixation is also linked to additional complications: nonabsorbable sutures can cause microabscesses and granuloma formation, leading to chronic testicular pain, while absorbable sutures usually result in minimal adhesions at the fixation site but may increase the risk of recurrent torsion.^17^

When diagnosis or treatment is delayed, possible complications include torsion, testicular ischemia, diffuse seminiferous tubule atrophy, marked impairment of spermatogenesis, and both acute and chronic pain 14, 18.

Infertility can occur due to increased testicular temperature, leading to a reduction in spermatogonia and spermatids with a relative increase in Sertoli cells.^11^^,^^13^ Therefore, early diagnosis and timely intervention are crucial to preserve testicular function and reduce the risk of malignant transformation.^13^

In the reported case, the presence of a pelvic bone fracture led to the testicular dislocation being overlooked, resulting in a delayed presentation to our setting.

Although the patient presented late, timely intervention after admission resulted in successful management. He was discharged from our clinic on the fourth postoperative day and was followed up in the outpatient clinic at two, four, and eight weeks after discharge. The patient showed significant clinical improvement, reported no new complaints, and was subsequently discharged from follow-up.

## 4 Conclusion

Testicular dislocation should be suspected in trauma patients presenting with inguinal pain, swelling, or pelvic fractures.

## Methods

The study has been reported in line with SCARE criteria.

## CRediT authorship contribution statement

**Wondwose Mengist Dereje:** Writing – review & editing, Writing – original draft, Visualization, Validation, Supervision, Formal analysis, Data curation, Conceptualization. **Misganaw Degu Worku:** Writing – review & editing, Writing – original draft, Supervision, Investigation. **Desalegn Kefale Aegash:** Writing – review & editing, Writing – original draft, Investigation. **Rahel Wubayehu Kahaliw:** Writing – review & editing, Writing – original draft, Investigation. **Mesafint Eyayu Tegegne:** Writing – review & editing, Writing – original draft, Investigation. **Biruktawit Abebe Assmamaw:** Writing – review & editing, Writing – original draft.

## Author agreement

We, the undersigned authors, transfer the copyright of this manuscript to the publisher and agree to abide by the journal's publication policies.

## Ethical statement

Ethical approval for this study was provided by the Ethical Committee of our institution on August 2025 under 863/25.

## Consent for publication

Written informed consent was obtained from the patient's parents for publication and any accompanying images. A copy of the written consent is available for review by the Editor-in-Chief of this journal on request.

## Funding

No funding

## Conflicts of interest

All authors declare that they have no conflict of interest.
